# Engineering of a novel cellulose-adherent cellulolytic *Saccharomyces cerevisiae* for cellulosic biofuel production

**DOI:** 10.1038/srep24550

**Published:** 2016-04-15

**Authors:** Zhuo Liu, Shih-Hsin Ho, Kengo Sasaki, Riaan den Haan, Kentaro Inokuma, Chiaki Ogino, Willem H. van Zyl, Tomohisa Hasunuma, Akihiko Kondo

**Affiliations:** 1 Department of Chemical Science and Engineering, Graduate School of Engineering, Kobe University, 1-1 Rokkodai, Nada-ku, Kobe 657-8501, Japan; 2 Organization of Advanced Science and Technology, Kobe University, 1-1 Rokkodai, Nada-ku, Kobe 657-8501, Japan; 3 State Key Laboratory of Urban Water Resource and Environment, School of Municipal and Environmental Engineering, Harbin Institute of Technology, Harbin 150090, PR China; 4 Department of Biotechnology, University of the Western Cape, Bellville 7530, South Africa; 5 Department of Microbiology, University of Stellenbosch, Stellenbosch 7600, South Africa; 6 Biomass Engineering Program, RIKEN, 1-7-22 Suehiro-cho, Tsurumi-ku, Yokohama, Kanagawa 230-0045, Japan

## Abstract

Cellulosic biofuel is the subject of increasing attention. The main obstacle toward its economic feasibility is the recalcitrance of lignocellulose requiring large amount of enzyme to break. Several engineered yeast strains have been developed with cellulolytic activities to reduce the need for enzyme addition, but exhibiting limited effect. Here, we report the successful engineering of a cellulose-adherent *Saccharomyces cerevisiae* displaying four different synergistic cellulases on the cell surface. The cellulase-displaying yeast strain exhibited clear cell-to-cellulose adhesion and a “tearing” cellulose degradation pattern; the adhesion ability correlated with enhanced surface area and roughness of the target cellulose fibers, resulting in higher hydrolysis efficiency. The engineered yeast directly produced ethanol from rice straw despite a more than 40% decrease in the required enzyme dosage for high-density fermentation. Thus, improved cell-to-cellulose interactions provided a novel strategy for increasing cellulose hydrolysis, suggesting a mechanism for promoting the feasibility of cellulosic biofuel production.

As the demand for fossil fuels increases and atmospheric CO_2_ levels continue to rise, biofuels have attracted increasing attention as sustainable and renewable alternative energy sources^1^. Lignocellulose is a potential resource for biofuel generation because of its low cost and large-scale availability^2^. However, the need for high-dosages of costly commercial cellulases in the saccharification process makes it challenging for cellulose-based biofuels to be economically feasible^3^. Although two leading enzyme companies (Genencor and Novozymes) have significantly reduced cellulase prices (to 15–20 cents per gallon of ethanol produced), these prices are still 5- to 10-fold higher than those of the amylases used for starch-based biofuel production^4^. Thus, development of a microorganism that is capable of both producing cellulases and fermenting resultant sugars into biofuels is a promising approach to alleviate the economic burden imposed by the need for commercial enzymes^5^.

*Saccharomyces cerevisiae* has been reported to have a superior capacity for converting glucose into ethanol^6^. However, yeast lacks the cellulolytic enzymes needed to degrade cellulose into glucose, such as β-glucosidase (BGL), endoglucanase (EG), and cellobiohydrolase (CBH). Several engineered *S. cerevisiae* strains capable of producing heterologous cellulases have been reported^7^,^8^. However, these engineered strains are unable to degrade crystalline cellulose effectively (achieving only 8–23% hydrolysis efficiency)^9^,^10^. This is due to insufficient production (or secretion) of CBH^11^, as CBHs are responsible for the degradation of crystalline cellulose and have been considered key forces in disrupting the recalcitrant structure of cellulose^12^.

Up to now, cellulolytic *S. cerevisiae* strains have been constructed primarily via either secretion or cell-surface display^13^. Secretion system, which releases enzymes into extracellular environment, is the most common route of cellulase production in recombinant strains. Although free enzymes can easily penetrate into the secondary cell wall of plant cells^14^, they are incapable of being recycled usage during an industrial process^13^. In comparison, a cell-surface display system permits immobilization of enzymes on the cell surface through glycosylphosphatidylinositol (GPI) anchoring of proteins^15^. Immobilization of numerous cellulases on a given microbial cell provides an effective increase of local enzyme concentration; facilitates synergistic interactions among enzymes; and, most importantly, enables re-utilization of enzymes in repeated fermentation, improving the economic feasibility of the process^16^. The cellulose degradation mechanisms of free-form cellulases and cellulosomes (complexed cellulase system) have been studied intensively in the last few decades^14^,^17^, however, few investigations have focused on the mechanisms employed by cell-surface-displayed enzymes (non-complexed cellulase system). Cellulosic ethanol production using cellulase-displaying cells is gaining increased attention^16^, but information on how these cells digest cellulose to fermentable sugars is still unclear.

To achieve commercial scale, high bioethanol productivity will need to be obtained from lignocellulosic waste (*e.g.*, rice straw). However, most studies select only pure/model cellulose (*e.g.*, phosphoric acid swollen cellulose (PASC) or Avicel) as an experimental substrate^16^, a model that is significantly different to real word ethanol fermentation from lignocellulosic feedstock. Here, we report the successful engineering of a cellulose-adherent *S. cerevisiae* strain producing heterologous BGL, EG, CBH1, and CBH2 via cell-surface display, permitting direct ethanol production from a realistic lignocellulosic substrate (rice straw). The interactions between engineered yeast cells and cellulose were imaged by scanning electron microscopy (SEM) to demonstrate clearly the dynamic mechanisms of cell-to-cellulose adhesion. This work provided valuable information on how to increase cellulose hydrolysis efficiency by enhancing cell-to-cellulose interactions for various types of substrate, and therefore may lead to a feasible path towards the cost-competitive production of cellulosic ethanol.

## Results

### Heterologous expression of cellulases in *S. cerevisiae*

**Figure 1:**
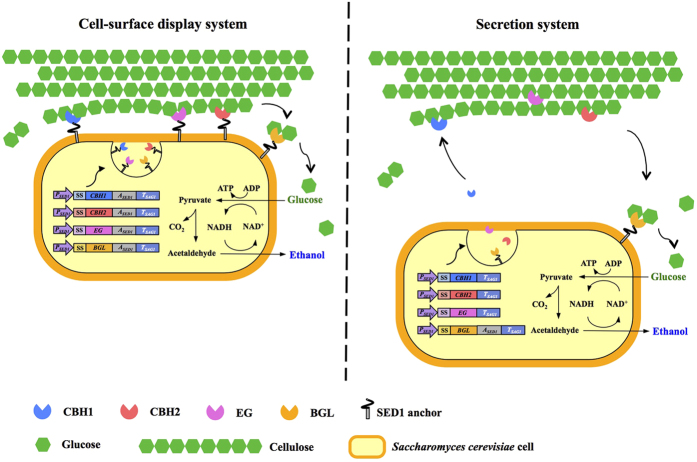
Engineering cellulolytic *S. cerevisiae* for cellulosic ethanol production via either cell-surface display or secretion of enzymes. *P*_*SED1*_, *SED1* promoter; SS, secretion signal; *A*_*SED1*_, *SED1* anchoring region; *T*_*SAG1*_, *SAG1* terminator.

The relevant features of the recombinant yeast strains used in this study are listed in Supplementary Table 1. Three codon-optimized genes that encode *Trichoderma reesei* EG2, *Talaromyces emersonii* CBH1, and *Chrysosporium lucknowense* CBH2 were expressed and used for assembly of enzyme cocktails in a BGL-displaying *S. cerevisiae* BY4741. Cellulase enzyme encoding genes were all expressed under the control of the *SED1* promoter, which is highly induced during the stationary-phase growth^18^. The secretion signal peptide of BGL1 was derived from *Rhizopus oryzae* glucoamylase, while EG2, CBH1, and CBH2 were produced with their native secretion signals. The schemes of engineered cellulolytic yeast strains producing enzymes via cell-surface display or secretion are illustrated in Fig. 1. In the strain expressing cell-surface-displayed proteins, the enzymes were encoded fused to the N-terminus of Sed1, a *S. cerevisiae* cell wall protein rich in threonine/serine residues that contains a putative GPI attachment signal. Sed1 becomes the most abundant cell wall protein during the stationary phase of growth^18^. The GPI-anchor is attached to the Sed1-enzyme chimeric proteins in the endoplasmic reticulum and subsequently transferred to the cell surface through an *S. cerevisiae* secretory pathway. Upon reaching outer layer of the cell wall, chimeric proteins are covalently bound to β-1,6-glucan via the GPI anchor^19^. In contrast, a chimeric protein lacking the Sed1 domain passes through cell wall and is released into the extracellular medium (secretion system). Metabolic burden often occurs due to the expression of heterologous protein genes, resulting in inhibition of cell growth^20^. However, in the present study, the growth rates of the recombinant strains were similar to those of the parent strain (Supplementary Fig. 1), suggesting that production of the heterologous enzymes did not impose an obvious metabolic burden.

### Assembly of high-efficiency enzyme cocktail

**Figure 2:**
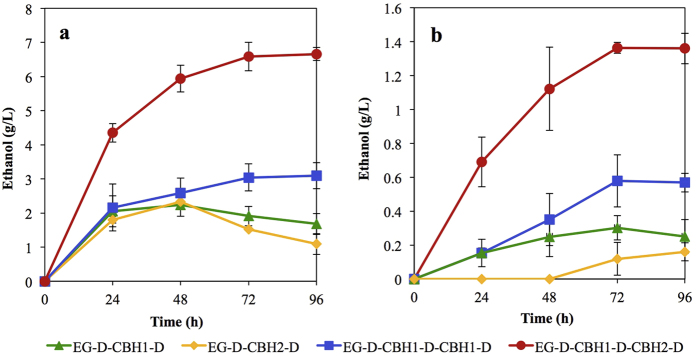
Time course of direct ethanol production from cellulosic materials. Ethanol was produced from 20 g/L PASC (**a**) and from 10 g/L Avicel (**b**). Data are presented as the means and standard deviations of triplicate measurements.

To develop a high-efficiency cellulase system, various cellulase combinations of some or all of our candidate proteins were engineered, including BGL, EG, CBH1, and CBH2. All enzyme gene combinations were expressed in *S. cerevisiae* and produced through cell-surface display technique, as depicted in Fig. 2. Cellulosic ethanol production from different recombinant strains was investigated as the indicator of cellulolytic activities in enzyme combinations. Pure celluloses consisting of PASC (amorphous type) or Avicel (crystalline type) were used as substrates. Two recombinant strains designated EG-D-CBH1-D and EG-D-CBH2-D (*i.e.*, containing combinations of BGL + EG + CBH1 and BGL + EG + CBH2, respectively) were designed as “minimum enzyme cocktails”. As shown in Fig. 2, a maximal ethanol production of 2.3 g/L (equivalent to 23% of the theoretical value) was achieved in both engineered strains using PASC as the substrate (Fig. 2a). In contrast, strain EG-D-CBH1-D showed a higher ethanol production rate than strain EG-D-CBH2-D when fermenting Avicel (Fig. 2b), suggesting that the *T. emersonii* CBH1 is more effective than *C. lucknowense* CBH2 in the degradation of crystalline cellulose.

Subsequently, an additional copy of the CBH1-encoding gene was added to BGL + EG + CBH1 cocktail, yielding strain EG-D-CBH1-D-CBH1-D. The transcription level of *CBH1* in strain EG-D-CBH1-D-CBH1-D was approximately 1.8-fold that of strain EG-D-CBH1-D (Supplementary Fig. 2). In PASC and Avicel fermentations, strain EG-D-CBH1-D-CBH1-D produced ethanol at 3.1 g/L (30% of the theoretical value) and 0.56 g/L (11% of the theoretical value), respectively, corresponding to 1.3- and 1.9-fold the levels seen in strain EG-D-CBH1-D (Fig. 2). To further increase the cellulose degradation efficiency, a strain (designated EG-D-CBH1-D-CBH2-D) that contained genes encoding BGL, EG, CBH1, and CBH2 was designed; this strain generated an ethanol titer of 6.7 g/L from PASC (66% of the theoretical value) and 1.4 g/L from Avicel (27% of the theoretical value), which are 2.9-fold and 4.5-fold higher than strain EG-D-CBH1-D. These results suggested that introduction of a second copy of the CBH1-encoding gene only slightly improved the cellulolytic activities, while the combination of both CBH1- and CBH2-encoding loci yielded a greater enhancement in the efficiency of cellulose hydrolysis.

### Performance of enzyme-secreting and -displaying cells in cellulose degradation

**Figure 3:**
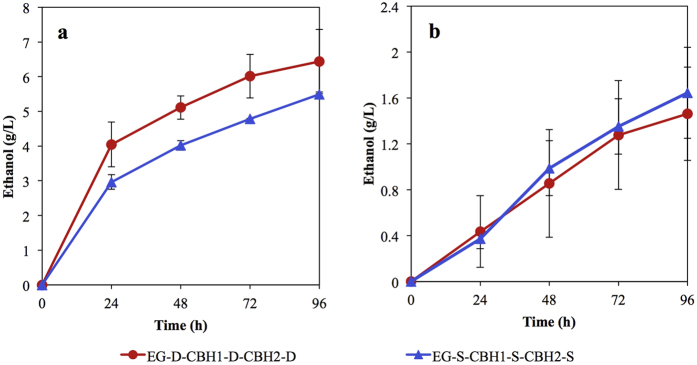
Time course of direct ethanol production using recombinant strains EG-D-CBH1-D-CBH2-D and EG-S-CBH1-S-CBH2-S. Ethanol was produced from 20 g/L PASC (**a**) and from 10 g/L Avicel (**b**). Data are presented as the means and standard deviations of triplicate measurements.

**Figure 4:**
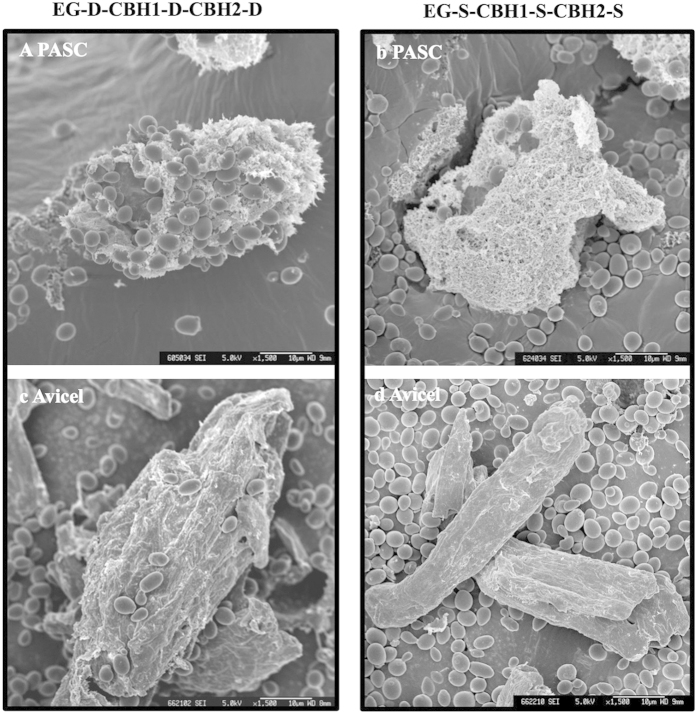
SEM micrographs of the interactions between cellulolytic *S. cerevisiae* cells and cellulosic materials. Interactions with PASC (**a**,**b**) and with Avicel (**c**,**d**). Cellulolytic cells (30 g/L) were incubated with 1% cellulosic materials (PASC or Avicel) for 2 h, and the cellulosic materials were used for SEM imaging. Scale bars are 10 μm.

**Figure 5:**
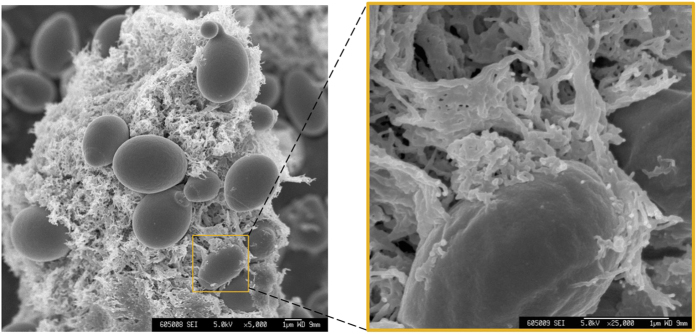
SEM micrographs of the interactions between PASC and EG-D-CBH1-D-CBH2-D cells. The observation conditions were the same as those used in Fig. 4. Scale bars are 1 μm.

We next compared the cellulose fermenting efficacy of *S. cerevisiae* strains expressing the EG, CBH1, and CBH2 cellulases via either cell-surface display or secretion. As depicted in Fig. 3a, the cellulase-displaying cells (EG-D-CHB1-D-CBH2-D) showed better ethanol yield (63% of the theoretical yield) from PASC than was seen from cellulase-secreting cells (EG-S-CBH1-S-CBH2-S, 54% of the theoretical yield), which is in good agreement with previous studies^21^. Based on the SEM observation shown in Fig. 4a, PASC appeared to form a more sponge-like surface material than Avicel. After incubation of yeast cells with cellulose, numerous cellulase-displaying cells (EG-D-CBH1-D-CBH2-D) remained attached to the PASC surface, while very few cellulase-secreting cells (EG-S-CBH1-S-CBH2-S) were retained on the cellulose surface (Fig. 4b). Notably, the adhesion between displayed cells and sponge-like cellulose (PASC) was clearly observed in Fig. 5, showing that the displayed cells are tightly bound to cellulose filaments. Unlike the enzyme-secreting strain, which digests the cellulose via free-form enzymes (such that yeast cell need have no direct interaction with cellulose), the enzyme-displaying strain apparently mediates cellulose degradation via attachment to the cellulose surface, which presumably significantly shortens the distance between microbe and substrate. However, interestingly, no obvious difference in ethanol titer between enzyme-displaying and -secreting cells was observed in Avicel fermentation (around 32% Avicel conversion, Fig. 3b), which may reflect lower binding efficiency of displayed cells to Avicel than to PASC (Fig. 4). We hypothesize that rough, sponge-like structure of PASC provides greater surface area and fine structures, thereby facilitating adhesion of displaying cells and promoting degradation of the substrate. To our knowledge, this is the first report on observation of the adhesion between cellulolytic yeast cells and cellulose.

To better understand the dynamic degradation mechanisms employed by displaying or secreting cells, the morphological changes of celluloses after 24 h degradation were monitored (Supplementary Fig. 3). Cellulose fibers (both PASC and Avicel) were observed to be disrupted into small, irregular pieces that attached with the displaying cells. In contrast, the cellulose fibers digested by secreting cells appear to be evenly ablated from the outer layer of microfibers, which is consistent with a previous report^14^. Taken together, these results indicated that the cellulase-displaying strain employs a “tearing” mechanism distinct from the ablative mechanism used by free cellulases to break down cellulose, such that enzyme-displaying cells seemed in purpose of achieving greater substrate surface area, thereby facilitating cell-to-cellulose adhesion, which is expected to enhance ethanol production.

### Targeted pre-treatment of rice straw

**Figure 6:**
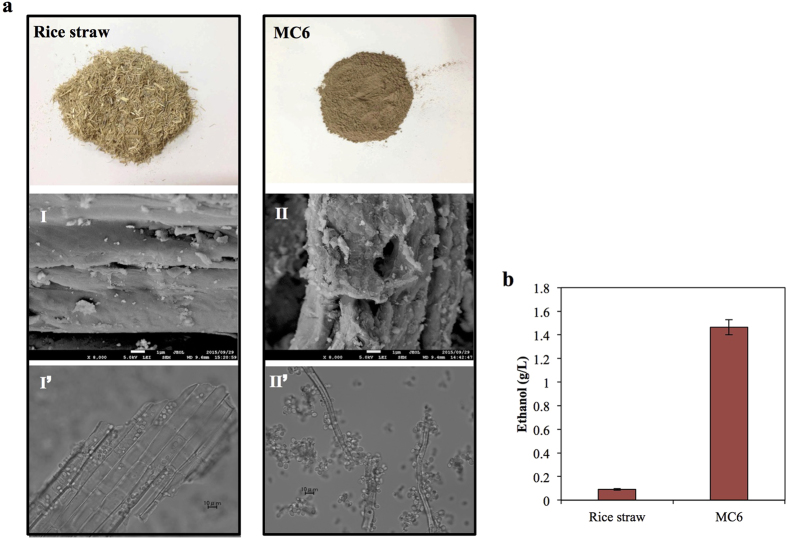
Comparison of cellulosic feedstock rice straw with MC6 for cellulosic ethanol production. (**a**) SEM micrographs of the surface structures on cellulosic materials (**I**,**II**); observation of adhesion between strain EG-D-CBH1-D-CBH2 and cellulosic materials using optical microscopy (**I’**,**II’**). (**b**) Ethanol yields at 96 h of fermentation from 25 g/L rice straw and MC6, respectively. Data are presented as the means and standard deviations of triplicate measurements.

To verify our hypothesis, that higher ethanol yield can be obtained using rough, sponge-like cellulose as a substrate that provides more surface area and finer structure for adhesion by enzyme-displaying yeast cells, the surface properties of cellulose obtained from agricultural waste (specifically, from rice straw) were altered. Liquid hot water (LHW) pretreatment has been reported to disrupt recalcitrant microstructure and remove 35–60% of the lignin and all of the hemicellulose from lignocellulose materials^22^; separately, milling has been reported to reduce particle size and crystallinity of lignocellulose, resulting in increased surface area for enzymatic attack^23^. In the present study, rice straw was treated with LHW plus 4 cycles of milling, and the resultant biomass was designated MC6. The morphological characteristics of rice straw and MC6 were observed by SEM (Fig. 6aI,II). Unprocessed rice straw exhibited a flat, rigid, and compact surface, while the surface of MC6 was rough and sponge-like. The average diameters of MC6 particles were about 20- to 50-fold smaller than those of rice straw. These results suggested that LHW and milling pretreatment significantly altered the surface characteristics of rice straw into a more sponge-like structure with increased surface area.

It was also observed that more cellulolytic cells adhere to MC6 fibers than to unprocessed rice straw (Fig. 6aI’, II’), a result that correlated with the obtained ethanol concentrations (Fig. 6b). These results suggested that rough, sponge-like cellulose particles facilitated cell-to-cellulose adhesion, thereby improving cellulose degradation.

### Evaluating the feasibility of high-density cellulosic ethanol production

**Figure 7:**
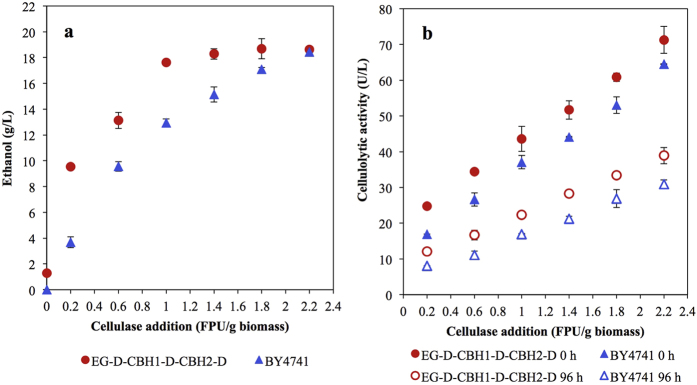
Evaluations of commercial cellulase addition in ethanol production from 100 g/L MC6. (**a**) Ethanol production at 96 h of fermentation with the addition of 0, 0.2, 0.6, 1.0, 1.4, 1.8, and 2.2 FPU/g-biomass cellulase (C-Tec2). (**b**) Cellulolytic activities in the fermentation media at 0 and 96 h of fermentation with the respective strains. Data are presented as the means and standard deviations of triplicate measurements.

To test the feasibility of high-density cellulosic ethanol production using strain EG-D-CBH1-D-CBH2-D, fermentation was conducted using 100 g/L MC6 as the substrate. Only 1.3 g/L ethanol (7% of the theoretical yield) was produced by enzyme-displaying cells after 96 h fermentation, while no ethanol was obtained by wild type strain (BY4741). Considering the low ethanol yields in both two strains (Fig. 7a), the inclusions of small amounts of exogenous commercial enzyme (C-Tec2) were still required to support hydrolysis of MC6. To address the economic feasibility of fermentation of MC6 using the enzyme-displaying strain, the potential diminution of C-Tec2 supplementation by using displaying cells was assessed. As shown in Fig. 7a, the ethanol yield from EG-D-CBH1-D-CBH2-D fermentation of MC6 was 7-fold increased by addition of trace amount of exogenous C-Tec2 (0.2 FPU/g-biomass). Specifically, the inclusion of 1.0 FPU/g-biomass C-Tec2 together with displaying cells provided a high cellulosic ethanol yield of 18 g/L, equivalent to 80% of the theoretical value. The result represents an approximately 44% decrease of the required enzyme dosage compared to that required for M6 fermentation by the wild-type strain. Additionally, although approximately half of the C-Tec2 activity was lost after 96 h of fermentation, the cellulolytic activities from displaying cells (indicated as the activity difference between EG-D-CBH1-D-CBH2-D and BY4741 fermentations) persisted (Fig. 7b), demonstrating the feasibility of recycling of cellulase-displaying yeast cells.

## Discussion

One of the main bottlenecks impeding the widespread production of cellulosic bioethanol is the large amount of costly commercial enzymes required for hydrolysis of cellulose^5^. As described in this work, a cellulose-adherent *S. cerevisiae* that displayed BGL, EG, CBH1, and CBH2 on the cell surface was constructed through a series of rational designs. It was demonstrated that the cellulase-displayed yeast strain employed a cell-to-cellulose adhesion and a “tearing” pattern as parts of its cellulose-degradation mechanism, which differed from those of strains producing free-form enzyme. Appropriate pretreatment of lignocellulose materials aiming to enhancing cell-to-cellulose interactions dramatically enhanced cellulosic ethanol yield. The resultant cellulose-adherent *S. cerevisiae* may significantly reduce the need for exogenous enzyme, potentially alleviating the bottleneck in commercial production of cellulosic bioethanol.

A crucial point in assembly of the cellulase cocktail is to maximize the synergistic actions among enzymes. Both CBH1 and CBH2 are known to act as exoglucanases, initiating cleavage of cellulose chains from reducing and non-reducing ends, respectively^24^. In this study, *T. emersonii* CBH1 appeared more important than *C. lucknowense* CBH2 in the synergism with BGL and EG (especially in Avicel fermentation), but we found that providing both CBH1 and CBH2 in enzyme cocktail was more effective on improving cellulolytic activities than increasing the proportion of CBH1 (here, by doubling the gene dose of the CBH1 encoding locus). This is because CBH2 can synergistically enhance the hydrolysis efficiency of CBH1, that it diminished the bumpy surface on cellulose, apparently preventing CBH1 from getting stuck during processive movement^25^. To date, a maximum of three kinds of cellulases (non-complexed cellulase system) have been simultaneously displayed on one single cell, because of the difficulty in displaying stable and functional heterologous proteins on the microbial surface. In the present work, we provided the first report (to our knowledge) of tethering four types of heterologous cellulases to the surface of a single yeast cell. The resulting strain exhibited significantly higher ethanol yield from both amorphous cellulose and crystalline cellulose compared to the cellulolytic yeast strains previously described in relevant studies.

Most reports have assessed enzyme production strategies using amorphous cellulose as substrate, and the cellulosic ethanol yield obtained with cell-surface display systems was generally higher than that obtained with secretion systems^26^,^27^. However, we proved that the performance of each system is actually substrate-dependent. The higher ethanol yields in enzyme-displaying systems are obtained only using amorphous cellulose as substrate, indicating that there is likely unique interactions between displaying cells and substrate, differing from those that occur in secreting cell. Cellulase-displaying cells degrade cellulose via tight attachment onto cellulose filaments, while cellulase-secreting cells did not exhibit obvious interactions with substrate. One of the reasons for the cell-to-cellulose adhesion is the high affinity of the carbohydrate binding domain (CBD) in cellulase towards cellulose^28^, and anchoring cellulase on cell surface results in a higher affinity of cells towards cellulose. Increased adhesion of *Escherichia coli* to cotton fibers has been demonstrated via anchoring the CBD from CBH onto the cell surface^29^. We also found that the adhesion between displaying cells and cellulose correlates with the hydrolysis efficiency of cellulose. Electron microscopy (Fig. 5) clearly illustrated that the swollen, rough microfibers of PASC can encircle the cellulase-displaying cells, thereby enhancing the adhesion between cells and cellulose, which may explain the higher ethanol yield obtained from PASC using displaying cells. The cellulose-adherent characteristic of enzyme-displaying cells is expected to shorten the distance between cells and substrates, thereby facilitating the mass transfer of hydrolysis products, particularly in high-density fermentation.

Notably, two distinct cellulose-degradation mechanisms are proposed based on the morphological changes of cellulose caused by both displayed and secreted cells (Supplementary Fig. 3). The results indicated that the cell-surface display system tends to increase the accessible surface area for cell-adhesion by tearing microfibers from cellulose particles. In contrast, free (secreted) enzymes equably erode cellulose surface and diminish particle size, consistent with a previous report that free enzymes employ an ablative, fibril-sharpening mechanism during cellulose degradation^30^. A potential explanation for the different mechanisms is the distinct enzyme-to-substrate interactions represented by the two systems. It has been known that free enzymes repeatedly associate and dissociate with cellulose to avoid getting stuck during processive movement^31^. In contrast, owing to cell-to-cellulose adhesion, the processive movements of surface-displayed enzymes are slowed down; enzymes are entrapped on cellulose and digestion proceeds without repeated cycles of association and dissociation with the substrate. As a result, the displayed cellulases are more likely to carry out deeper directed digestion of cellulose particles than are free enzymes, resulting in the splayed morphology observed in Supplementary Fig. 3.

Natural lignocellulose (*e.g.*, rice straw) is usually rigid and lacks the rough, intricate surface structures presented in PASC. Thus, to increase the adhesion between natural lignocellulose and cellulase-displaying yeast, the surface structures of rice straw were broken into more “sponge-like” materials via pretreatment by LHW and milling. In our work, cellulase-displaying yeast cells preferred to attach to MC6 and exhibited dramatically higher hydrolysis rates than those seen with untreated rice straw, presumably because the MC6 has more surface area with favorable sites for cell-to-cellulose adhesion. Thus, appropriate pretreatment of lignocellulose, which adopts the cell-to-cellulose adhesion pattern in combination with cellulase-displaying yeast, is expected to effectively promote the degradation efficiency. Although the optimal biomass pretreatment combined with free-cellulase-mediated saccharification has been extensively studied^23^, the pretreatment process suitable for surface-displayed cellulases still remains obscure. Pretreatment by steam explosion has been reported to be capable of producing pores with 3-nm to 1-μm diameters on the surface of lignocellulose^32^, facilitating the access of free enzymes (approximately 5.1 nm in diameter) to the interior of cellulose particles^33^. However, the enzyme-displaying cells (around 3 μm) would not be able to enter pores of this size; such cells would instead be expected to digest the substrate primarily by “shaving” cellulose fibers from the external surface of substrate particles. As a result, such a “pore-punching” pretreatment process may be not ideal for cell-surface display hydrolysis systems. On the contrary, the method of ammonia fiber expansion (AFEX) pretreatment can effectively increase the surface roughness of lignocellulose materials^34^, which likely will be of greater use in combination with cell-surface display systems.

Natural lignocellulose is commonly considered as the most rigid substrate resistant to digestion, and impossible to degrade by cellulolytic yeast strains in the absence of exogenous enzymes. Even with addition of exogenous enzymes, natural lignocelluloses rarely provide high ethanol yields (over 80% of theoretical value) when used as the substrate for simultaneous saccharification and fermentation (SSF)^35^. In the reported SSF for bioethanol production mediated by *S. cerevisiae*, cellulase loadings of 15–30 FPU/g-biomass are generally used, depending on the specific substrate^36^,^37^. Recently, Matano *et al.* reported the use of cellulase-displayed yeast strain to diminish cellulase dosage to 10 FPU/g-biomass in ethanol production from LHW-pretreated rice straw^38^. Surprisingly, the engineered cellulose-adherent *S. cerevisiae* in the present study is capable of converting pretreated rice straw into ethanol without commercial enzyme addition. To our knowledge, this is the first report of direct ethanol production from nature lignocellulose using a cellulolytic yeast strain, presenting a new potential towards making efficient use of lignocellulose. Moreover, nearly 80% of ethanol theoretical yield was achieved in the presence of only 1 FPU/g-biomass C-Tec2, a quantity that represents a 44% decrease of enzyme loading compared with that required in non-cellulolytic *S. cerevisiae* and a much lower enzyme dosage compared with other reports. Meanwhile, the enzymes immobilized on the cell surface appeared higher stability during the fermentation than did commercial enzymes. This observation is in agreement with the previous report that tethering of enzymes to solid supports can increase the protein stability under non-optimal reaction conditions such as low temperature and organic solvent composition^39^. The retention of activities in displayed enzymes also demonstrates the feasibility of using cell-surface display in cell-recycling processes. By constructing a novel cellulose-adherent *S. cerevisiae* with surface-displayed cellulases, we successfully demonstrated the concept of enhancing cell-to-cellulose interactions to effectively alleviate the requirement for high doses of supplemental enzymes in cellulosic ethanol production. Further improvement of cell-to-cellulose adhesion (*e.g.*, increased display of carbohydrate binding domains) combined with the increased cellulolytic activities on yeast surface is expected to provide a viable model for implementation of economically feasible cellulosic ethanol production.

## Methods

### Media and materials

*Escherichia coli* NovaBlue was grown in Luria-Bertani broth at 37 °C, and 100 mg/L ampicillin was added to the medium when required. Yeast strains were screened on synthetic dextrose (SD) agar plates (6.7 g/L of yeast nitrogen base without amino acids and 20 g/L of glucose) supplemented with appropriate amino acids. Yeast strains were pre-cultured at 30 °C for 72 h in yeast extract-peptone (YP) medium (10 g/L yeast extract and 20 g/L peptone) containing 20 g/L glucose (YPD). Cellulosic ethanol production was carried out at 37 °C in YP media containing different cellulosic materials depending on the purpose of different fermentation experiments. PASC was prepared from Avicel PH-101 (Fluka Chemie GmbH, Buchs, Switzerland) as described previously^7^. Rice straw was pretreated with a liquid hot water method (130–300 °C under a pressure of less than 10 MPa) and then subjected to 4 cycles of milling using a CMJ01 nano-mech reactor (Techno Eye, Tokyo, Japan)^40^; the resultant biomass was designated MC6. The composition of MC6 was 43% (w/w) glucan, 2% (w/w) xylan, 42.3% (w/w) ash and lignin, and 12.7% (w/w) other materials^41^.

### Plasmid and strain constructions

The plasmids and primers used in this study are summarized in Supplementary Table 1 and 2, respectively. The plasmid pRDH227 was constructed by removing the *C. lucknowense* open reading frame as a 1468 bp PacI/AscI fragment from the plasmid pMU784^11^ and cloning it into the corresponding sites of pBHD1^42^. Other plasmids were constructed by connecting DNA fragments using the isothermal assembly method^43^. For plasmid pDI9-CBH1_D_, four PCR products were connected. The four PCR products included the following: *I9* region fragments (the 3’ non-coding region between gene *YOR191W* and *YOR192C*, amplified from the genome of *S. cerevisiae* BY4741 using primers pairs I9a-M-F + I9a-O-R and I9b-O-F + I9b-C1-R; *ori*-*ampR* fragment (from plasmid pIU-CBH1_D_) using primers O-I9a-F + O-I9b-R; *MET15* fragment (from plasmid pRS401) using primers M-I9a-F + M-C1-R; and *T. emersonii* CBH1-encoding surface-display cassette (from plasmid pI5-CBH1_D_) using primers C1-M-F + C1-I9b-R. To construct plasmid pDI9-CBH2_D_, a PCR-amplified *C. lucknowense* CBH2-encoding gene (from plasmid pRDH227, using primers C2-F + C2-R) and the vector backbone containing the enzyme-display cassette (from plasmid pDI9-CBH1_D_, using primers D-C2-F + P-C2-R) were linked together. Similarly, the *CBH2* gene (primers C2-F and C2-R2) was ligated with the vector backbone containing the enzyme-secretion-cassette (from plasmid pDI9-CBH1_D_, using primers D-C2-F2 + P-C2-R), to yield plasmid pDI9-CBH2_S_. In addition, the *CBH2* gene, enzyme-display cassette, and *I5* region (the 3’ non-coding region of gene *YLL055W* and *YLL054C*, amplified from plasmid pI5-CBH1-D using primers D-C2-F + P-C2-R) were connected to yield plasmid pIU5-CBH2_D_.

Plasmids were transformed into *S. cerevisiae* BY4741 using lithium acetate as described^44^, and integrated into either the *I5* or *I9* region by homologous recombination. The transformants were identified using colony PCR to check for the integration of cellulase genes (using screening with primers I9-F + I9-R for *I9* region integration or primers I5-F + I5-R for *I5* region integration).

### Cell growth assay

To measure the growth of yeast cells, parent strain *S. cerevisiae* BY4741 and recombinant strains were cultivated individually in SD medium at 30 °C with shaking at 150 rpm for 24 h. The resulting pre-cultures were inoculated into 5 mL YPD medium at an initial optical density (OD_660_) of 0.05 and cultivated at 30 °C with shaking at 70 rpm. The value of the OD_660_ was measured once hourly using a TVS062CA Bio-photorecorder (Advantec Toyo, Tokyo, Japan). The value of the OD_660_ was taken as an indicator of cell growth.

### Quantitative real-time PCR

The transcription levels of the cellulase-encoding genes were quantified as described previously^45^. Primers rt-CBH1-F and rt-CBH1-R were used to determine the transcription level of the *CBH1* gene; primers rt-CBH2-F and rt-CBH2-R were used to determine the transcription level of the *CBH2* gene. Transcription levels of the target genes were normalized to those of the housekeeping gene *ACT1* (tested using primers rt-ACT1-R and rt-ACT1-F).

### Scanning electronic microscopy and optical microscopy

Yeast cells were resuspended in phosphate buffer (pH 5.0) at a concentration of 30 g wet cells/L. Subsequently, 1% (w/v) PASC or Avicel was added to the cell suspension, which then was incubated at 37 °C for 2 h. Cellulose fibers were fixed with 4% paraformaldehyde and 4% glutaraldehyde (GA) in 0.1 M cacodylate buffer (pH 7.4) at 4 °C. Thereafter, fibers were fixed with 2% GA in cacodylate buffer overnight. The samples were additionally fixed with 1% tannic acid at 4 °C for 2 h. After the fixation the fibers were washed with cacodylate buffer 4 times, followed by post fixation with 2% osmium tetroxide in cacodylate buffer for 4 h. The samples next were dehydrated in a graded series of ethanol solutions (50, 70, 90, and 100%), and then were substituted into tert-butyl alcohol and dried by vacuum freeze drying. After drying, the samples were coated using an osmium plasma coater (NL-OPC80NS, Nippon Laser & Electronics Laboratory, Nagoya, Japan). The samples were visualized using a scanning electron microscope (JSM-6340F; JEOL Ltd., Tokyo, Japan) at an acceleration voltage of 5 kV. For observation using optical microscopy, post-incubation samples were directly applied onto microscope slides and imaged.

### Ethanol production from cellulosic materials

Fermentations were performed at 37 °C under oxygen limited conditions at an agitation speed of 200 rpm in 100-mL closed bottles equipped with a bubbling CO_2_ outlet and a stir bar. Pre-cultivated cells in YPD medium were centrifuged and washed twice by sterilized water. The collected cells were inoculated into 20 mL YP medium containing 20 g/L PASC, 10 g/L Avicel, or 100 g/L MC6. Initial cell densities were adjusted to approximately 150 g wet cells/L. Yeast cell wet weight was determined by weighing a cell pellet that was harvested by centrifugation at 1,000 × g for 5 min. To evaluate cellulase dosages in the SSF process, commercial enzyme (Novozymes Cellic CTec2; Novozymes Inc., Bagsvaerd, Denmark) was added into medium at 0-h of fermentation at enzyme concentrations of 0, 0.2, 0.6, 1.0, 1.4, 1.8, 2.2 FPU/g-biomass. Measurement of the filter paper cellulase units (FPU) of CTec2 was based on the standard NREL analytical procedure^46^ performed at 37 °C. The ethanol concentrations in the fermentation medium were determined using a gas chromatograph, as described previously^47^.

### Cellulolytic activity assay

At the 0-h and 96-h time points of ethanol production from 100 g/L MC6, fermentation medium was assayed for cellulolytic activities. Cellulolytic activity represents the degradation ability of all enzymes present in the fermentation broth. Fermentation broth was added into 50 mM sodium citrate buffer (pH 5.0) containing a final concentration of 1% (w/v) MC6 and 100 mM methyl glyoxal (Nacalai Tesque, Inc., Kyoto, Japan); the methyl glyoxal prevented the assimilation of glucose by yeast cells^48^. The reaction was performed at 37 °C using a heat block (Thermo Block Rotator SN- 06BN; Nissin, Tokyo, Japan) with shaking at 35 rpm, and the supernatant was collected by centrifugation. The amount of glucose in the supernatant was determined by the Glucose CII kit (Wako Pure Chemical, Osaka, Japan). One unit of cellulolytic activity (expressed as U/L) was defined as the amount of enzyme needed to produce 1 μmoL of glucose per minute at 37 °C, pH 5.0.

## Additional Information

**How to cite this article**: Liu, Z. *et al.* Engineering of a novel cellulose-adherent cellulolytic *Saccharomyces cerevisiae* for cellulosic biofuel production. *Sci. Rep.* **6**, 24550; doi: 10.1038/srep24550 (2016).

## Supplementary Material

Supplementary Information
