# Supplementary material for: Engineering of a novel cellulose-adherent cellulolytic Saccharomyces cerevisiae for cellulosic biofuel production
Source: Sci Rep. 2016 Apr 15;6:24550. doi: 10.1038/srep24550 (PMC4832201; doi:10.1038/srep24550)
Supplement: Supplementary Information [file srep24550-s1.doc]

**Engineering of a novel cellulose-adherent cellulolytic *Saccharomyces cerevisiae* for cellulosic biofuel production**

Zhuo Liu, Shih-Hsin Ho, Kengo Sasaki, Riaan den Haan, Kentaro Inokuma, Chiaki Ogino, Willem H. van Zyl, Tomohisa Hasunuma, Akihiko Kondo

**Supplementary Figures**

**Supplementary Figure 1 | Time-course profiles of cell growth using parent strain BY4741 and recombinant yeast strains in YPD medium.** Each strain was inoculated in YPD medium to an initial OD_660_ of 0.05 and then cultured aerobically at 30 °C, 70 rpm, for 72 h. For each strain, data are presented as the mean ± SD from three independent experiments.

**Supplementary Figure 2 | Relative transcription levels of *CBHI* and *CBH2* genes in recombinant yeast strains.** Gene *ACT1* was used as the internal standard. The relative transcription levels are shown normalized to the level observed in strain EG-D-CBH1-D-CBH2-D, whose relative transcription level was defined as 1. For each strain, data are presented as the mean ± SD from three independent experiments.


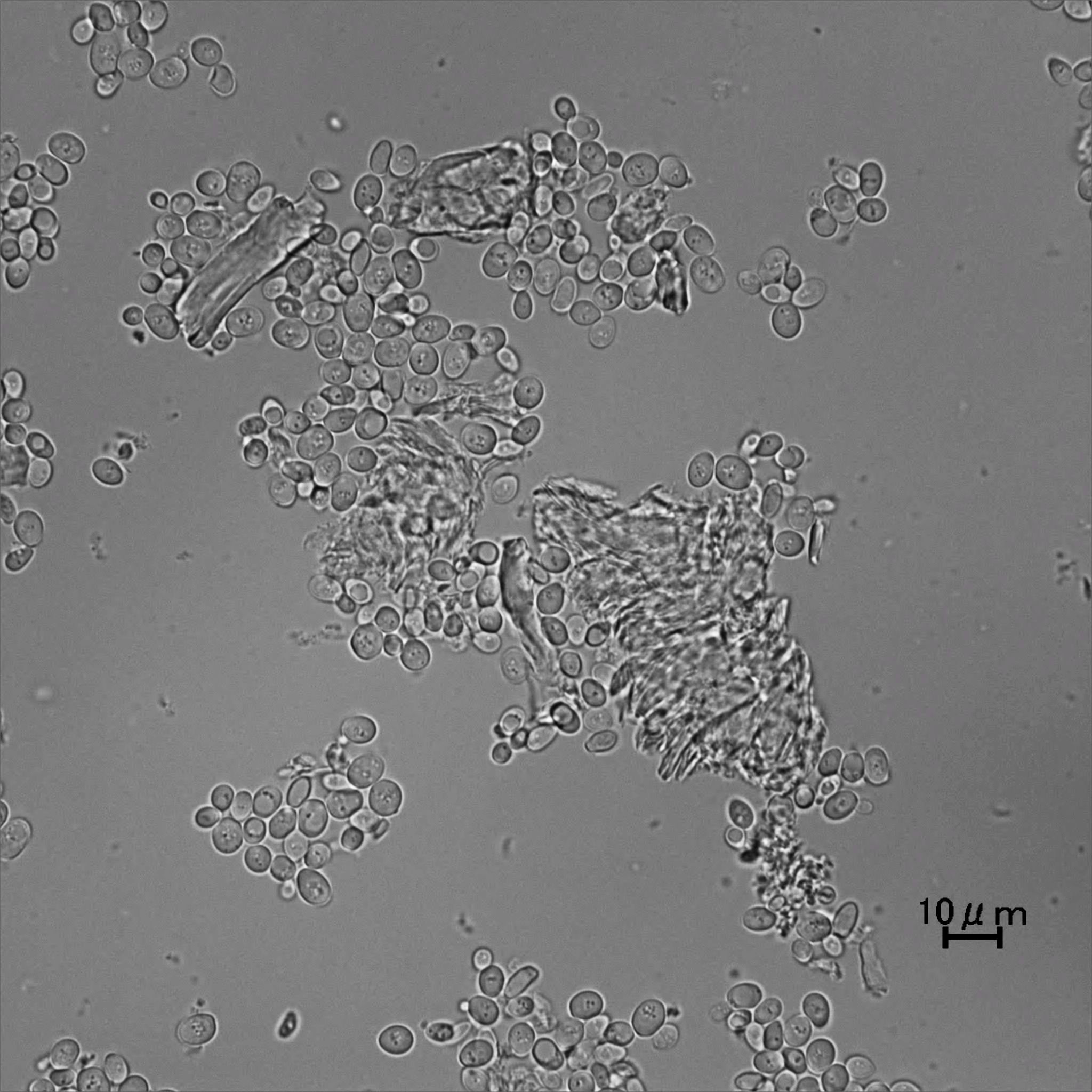

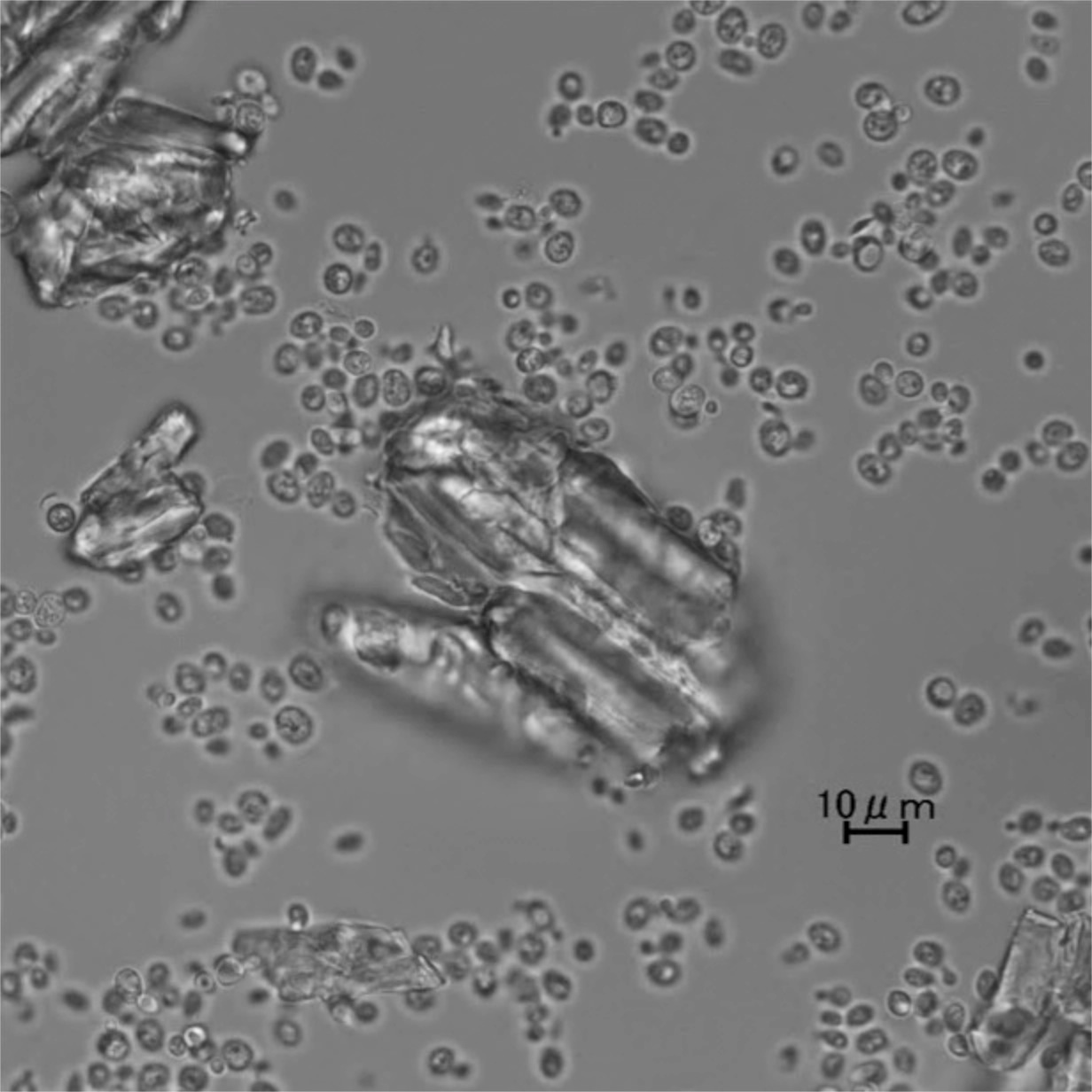

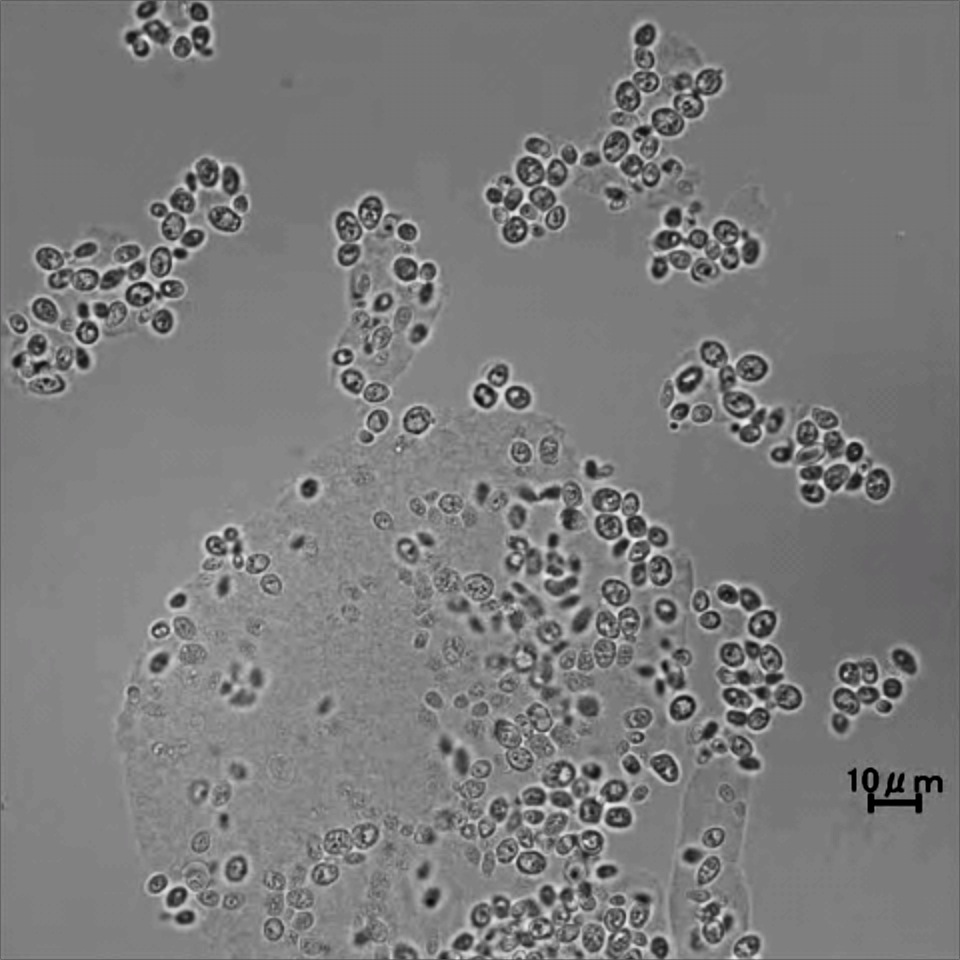

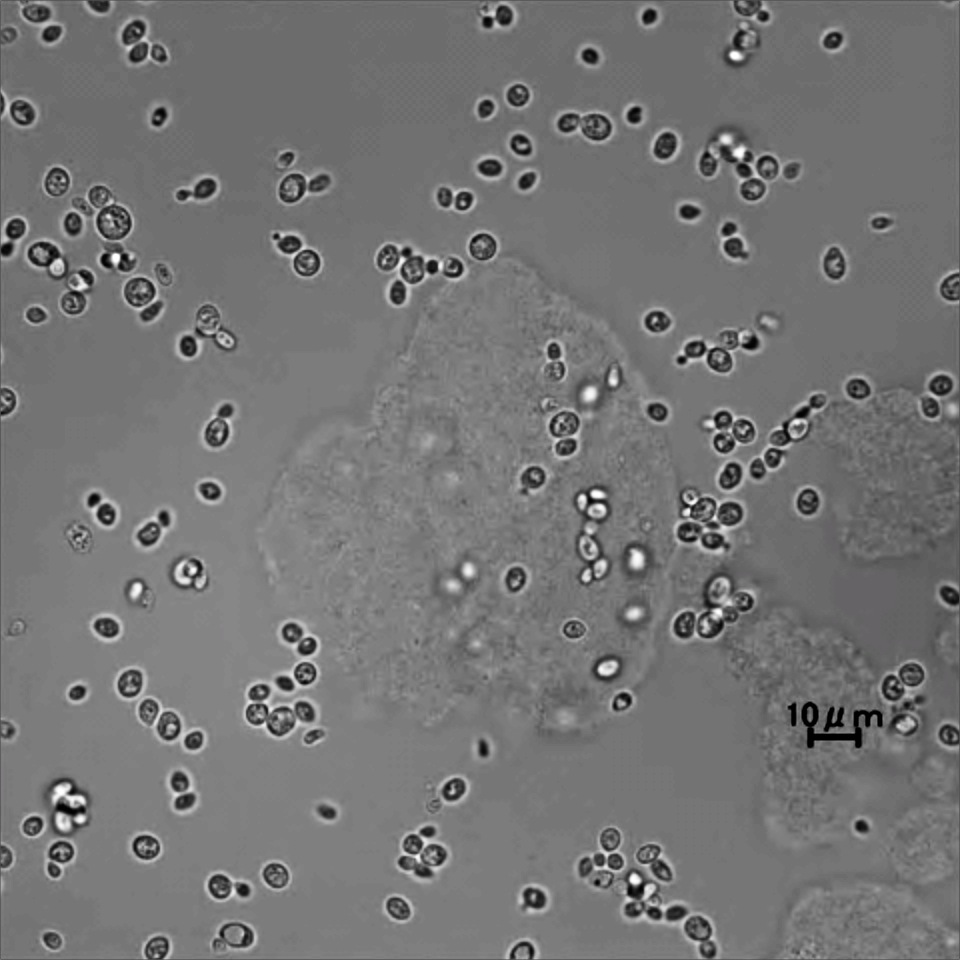


**a PASC**

**c Avicel**

**b PASC**

**d Avicel**

**EG-D-CBH1-D-CBH2-D**

**EG-S-CBH1-S-CBH2-S**

**Supplementary Figure 3 | Micrographs of PASC (a**, **b) and Avicel (c**, **d) degradation process by cellulolytic *S. cerevisiae*.** Cellulolytic cells (30 g/L) were incubated with 1% cellulosic materials for 24 h at 37 °C. Cellulosic materials were sampled and directly applied to microscope slides for observation. Scale bars are 10 μm.

**Supplementary Tables**

**Supplementary Table 1 | Yeast strains and plasmids used in this study**

| **Strains or plasmids** | **Description** | **Source** |
| --- | --- | --- |
| **Yeast strains** |  |  |
| BY4741 | *MAT***a** *his3Δ1 leu2Δ0 met15Δ0 ura3Δ0* | Life Technologies |
| EG-D | Display of BGL, EG | [32] |
| EG-D-CBH1-D | Display of BGL, EG, CBH1 | [32] |
| EG-S-CBH1-S | Display of BGL, secretion of EG, CBH1 | [32] |
| EG-D-CBH2-D | EG-D (pIU5-CBH2_D_), display of BGL, EG, CBH2 | This study |
| EG-D-CBH1-D-CBH1-D | EG-D-CBH1-D (pDI9-CBH1_D_), display of BGL, EG, CBH1 (two copies of *CBH1* genes) | This study |
| EG-D-CBH1-D-CBH2-D | EG-D-CBH1-D (pDI9-CBH2_D_), display of BGL, EG, CBH1, CBH2 | This study |
| EG-S-CBH1-S-CBH2-S | EG-S-CBH1-S (pDI9-CBH2_s_), display of BGL, secretion of EG, CBH1, CBH2 | This study |
| **Plasmids** |  |  |
| pRDH227 | *Hyg*, expression of *C. lucknowense CBH2* gene | This study |
| pIU5-CBH1_D_ | *URA3,* display of *T. emersonii* CBH1 | [32] |
| pIU5-CBH2_D_ | *URA3,* display of *C. lucknowense* CBH2 | This study |
| pDI9-CBH1_D_ | *MET15*, display of *T. emersonii* CBH1 | This study |
| pDI9-CBH2_D_ | *MET15*, display of *C. lucknowense* CBH2 | This study |
| pDI9-CBH2_s_ | *MET15*, secretion of *C. lucknowense* CBH2 | This study |

*C. lucknowense, Chrysosporium lucknowense; T. emersonii*, *Talaromyces emersonii*; BGL, β-glucosidase; EG, endoglucanase; CBH1, cellobiohydrolase 1; CBH2, cellobiohydrolase 2.

**Supplementary Table 2 | PCR primers used in this study**

| **Primers** | **Sequence (5’-3’)** |
| --- | --- |
| I9a-M-F | ATTAATGAATCGGCCAACGCTGGATATGACTGTGTTGTTGCTGATA |
| I9a-O-R | GGGGGCGGAGCCTATGGAAAAACGCCAGCAACGCGG |
| O-I9a-F | AAGGCCGCGTTGCTGGCGTTTTTCCATAGGCTCCGCCCCC |
| O-I9b-R | GCACTTTTCGGGGAAATGTGCGCGGAACCCCTATTTGTTTATTTTTC |
| I9b-O-F | AAACAAATAGGGGTTCCGCGCACATTTCCCCGAAAAGTGCCACC |
| I9b-C1-R | TTTTCACCGTCATCACCGAAGGGCCCATGGCTAGGTGT |
| M-I9a-F | ACACACCTAGCCATGGGCCCTTCGGTGATGACGGTGAAAA |
| M-C1-R | TTTCACACCGCATAGATCCGACTTGTGAGAGAAAGTAGGTTTAT |
| C1-M-F | ACCTACTTTCTCTCACAAGTCGGATCTATGCGGTGTGAAATAC |
| C1-I9b-R | CAACAACACAGTCATATCCAGCGTTGGCCGATTCATTA |
| C2-F | AATACGTTCGCTCTATTAAGATGGCCAAGAAGTTGTTCATTACC |
| C2-R | GTTGATAATTTACTCGAGCCGAATGGTGGATTTGCGTTCGTTAAC |
| C2-R2 | CTCAATGTACTAACTGTACATTAGAATGGTGGATTTGCGTTCG |
| D-C2-F | CGAACGCAAATCCACCATTCGGCTCGAGTAAATTATCAACTGTCC |
| P-C2-R | ATGAACAACTTCTTGGCCATCTTAATAGAGCGAACGTATTTT |
| D-C2-F2 | ACGCAAATCCACCATTCTAATGTACAGTTAGTACATTGAGTCTAAATA |
| I9-F | AAGAAGAAATCCGTGCTTACACATT |
| I9-R | GCTATCCCATGCAAAGATTGTCAACG |
| I5-F | CATTGAAGAAGGGAAAGTGGTAACC |
| I5-R | TCCCTCTCTAATCTGGGTGAGAC |
| rt-CBH1-F | CAACTTACTGTCCAGACGACGAAAC |
| rt-CBH1-R | AAGGAAGAACCAGAGGAGGTAACAC |
| rt-CBH2-F | AGAAGTCCCTAGTTTCCAATGGCTT |
| rt-CBH2-R | CGGCCTTATTCAAAGCTCTAACCTG |
| rt-ACT1-F | TGGATTCCGGTGATGGTGTT |
| rt-ACT1-R | TCAAAATGGCGTGAGGTAGAGA |
